# Heartwood/Sapwood Characteristics of *Populus euphratica* Oliv. Trunks and Their Relationship with Soil Physicochemical Properties in the Lower Tarim River, Northwest China

**DOI:** 10.3390/plants14020154

**Published:** 2025-01-07

**Authors:** Tongyu Chen, Tayierjiang Aishan, Na Wang, Ümüt Halik, Shiyu Yao

**Affiliations:** 1 College of Ecology and Environment, Xinjiang University, Urumqi 830046, China; chentongyu05@163.com (T.C.); wangna0819@163.com (N.W.); halik@xju.edu.cn (Ü.H.); ellayao1998@163.com (S.Y.); 2 Ministry of Education Key Laboratory of Oasis Ecology, Xinjiang University, Urumqi 830046, China

**Keywords:** *Populus euphratica*, heartwood/sapwood traits, soil nutrient dynamics, arid environment adaptation

## Abstract

The characteristics of heartwood and sapwood not only reflect tree growth and site quality but also provide insights into habitat changes. This study examines the natural *Populus euphratica* Oliv. forest in the Arghan section of the lower Tarim River, comparing the heartwood and sapwood characteristics of *P. euphratica* at different distances from the river, as well as at varying trunk heights and diameters at breast height (DBH). The objective was to examine the correlation between these characteristics and the physicochemical properties of the soil to better understand the ecological response strategies of *P. euphratica* in arid environments. Results indicated that heartwood radius, sapwood width, sapwood area, and heartwood moisture content decreased with increasing trunk height, following the pattern: 0.3 m > 0.8 m > 1.3 m. In contrast, heartwood density increased as trunk height increased. Most of the heartwood and sapwood indicators increased with larger tree diameters. In the case of *P. euphratica* with a DBH of less than 45 cm, the difference in moisture content between heartwood and sapwood was not significant (*p* > 0.05) at heights of 0.3 m and 0.8 m. However, at a height of 1.3 m, the difference was significant (*p* < 0.05). Soil analysis revealed that factors such as total nitrogen, available potassium, and water content significantly influenced the physical characteristics of *P. euphratica* heartwood and sapwood across different sites. Redundancy analysis (RDA) further demonstrated that total nitrogen, available phosphorus, and soil moisture were significantly correlated with the physical properties of *P. euphratica* heartwood and sapwood, further validating the critical role of soil nutrients in shaping the wood characteristics of *P. euphratica*. These findings highlighted the specific adaptations of *P. euphratica* in the lower Tarim River to the arid desert environment, reflected in the observed relationships between soil conditions and the physical characteristics of heartwood and sapwood.

## 1. Introduction

Forests, one of the most complex and diverse ecosystems on Earth, play a crucial role in maintaining global ecological balance and biodiversity. They provide habitats for understory species and contribute significantly to soil and water conservation [1]. Trees are integral to the maintenance of ecosystem diversity and stability, serving as the foundation for forest ecosystem structure and functioning. The growth status of a tree and its adaptive strategies are reflected in the trunk. In many species, heartwood and sapwood can be distinguished by color, with heartwood typically darker and sapwood lighter [2]. However, in some species, color alone may not reliably differentiate the two, and in certain environmental conditions, the color differences between heartwood and sapwood may be subtle or absent. For example, in some tree species, such as *Populus euphratica*, the color contrast is more pronounced, but this is not a universal characteristic. Sapwood and heartwood, the two primary components of the woody tissue, play critical roles in tree growth. Sapwood functions as the active zone for water and nutrient transport, while heartwood primarily provides structural support and stores nutrients and water [3,4]. The density of both tissues reflects the hydraulic properties of the xylem, while their sensitivity to environmental changes reveals the tree’s adaptive capacity in terms of long-term structural integrity and resource storage [5]. While both sapwood and heartwood contribute to the tree’s overall functionality, heartwood is generally less phenotypically adaptable to environmental fluctuations compared to sapwood, which is more directly involved in physiological processes such as water and nutrient transport. Nonetheless, the formation and density of both heartwood and sapwood are influenced by environmental factors, particularly water availability, which is crucial for the tree’s growth and survival in varying conditions.

*Populus euphratica* Oliv. (*P. euphratica*), also referred to as the heterophyllous poplar, is a deciduous tree belonging to the Salicaceae family and a prototypical representative species of the arid zone. It plays an irreplaceable role in maintaining the regional ecological balance and preventing desertification [6]. The growth of *P. euphratica* in arid zones is constrained by soil moisture. The physicochemical properties of soil are influenced by variations in moisture content at different distances from rivers, which significantly affects plant growth and their ability to absorb and utilize water and nutrients. These changes may lead to variations in the characteristics of both heartwood and sapwood, depending on environmental conditions and physiological factors. Such adaptations enable *P. euphratica* to survive in harsh desert environments [4,5,7,8,9]. Investigating the relationships between heartwood and sapwood characteristics, and soil physicochemical properties, will provide deeper insights into the ecological strategies of *P. euphratica* for adapting to arid environments.

Under arid conditions, the growth of *P. euphratica* xylem is primarily constrained by the availability of moisture. The formation of xylem is slowed and overall growth is reduced in areas with lower moisture contents [10]. Studies have shown that the heartwood and sapwood characteristics of *P. euphratica* exhibit both axial and radial differences. Radial growth is closely linked to heartwood formation. As the diameter at breast height (DBH) increases, the proportion of heartwood increases, whereas the proportions of sapwood and bark decrease. As trees mature, their internal structure undergoes significant changes. Heartwood gradually becomes the dominant tissue, providing essential structural support and stability, while sapwood, primarily responsible for water transport, becomes less prominent with age and increasing tree size. This shift in the relative proportions of heartwood and sapwood reflects the growing demand for structural strength as the tree matures, with the xylem transitioning from its primary role in water transport to supporting the tree’s structural integrity [11]. Heartwood formation is positively correlated with DBH and tree age, increasing with age but decreasing with height. Conversely, the proportion of sapwood decreases with age and is influenced by the tree’s physiological need for more developed heartwood to support its growing size and reduced reliance on sapwood for water transport at later stages [12]. This indicates that radial growth has a significant effect on heartwood and sapwood formation [13].

In recent years, the combined impacts of climate change and human activities have resulted in widespread degradation of the natural habitat of *P. euphratica*, leading to significant mortality of the trees [14,15]. As a result, the restoration and conservation of *P. euphratica* in extremely arid regions is an urgent priority. While some studies have focused on the functional traits of *P. euphratica* and the impact of environmental factors on its growth [10,16], there is still limited understanding of how soil physicochemical properties at varying distances from the river influence heartwood and sapwood characteristics. Moreover, the key soil indicators responsible for these changes remain poorly defined. This study, therefore, focused on *P. euphratica* forests in the lower reaches of the Tarim River, examining sites at different distances from the river channel. The aim was to investigate variations in heartwood and sapwood traits of *P. euphratica* in relation to associated soil physicochemical factors across these sites. The objective was to elucidate the intrinsic relationships between heartwood and sapwood traits and soil properties, providing scientific insights to support the ecological protection and sustainable management of *P. euphratica* forests.

## 2. Results

### 2.1. Physical Characteristics of Heartwood and Sapwood in Sample Tree Trunks

As illustrated in Table 1, the variation patterns of various indicators for heartwood and sapwood in *P. euphratica* across different diameter classes and heights are distinct. On average, the values for HR, SW, HA, SA, and HMC show a decreasing trend as one moves upwards from the base of the tree (0.3 m), with the mean values for heartwood generally higher than those for sapwood. In contrast, HD, SD, H%, and S% tend to decrease as height increases from the base, while SMC initially decreases and then increases as height progresses upward.

The variation trends of heartwood and sapwood indicators across different diameter classes are as follows: For diameter classes ranging from 15–25 cm, HD at 1.3 m height shows a significant difference from the other two heights (*p* < 0.05); SD at 0.8 m shows significant differences from the other two heights (*p* < 0.05); and HMC shows significant differences at all three heights (*p* < 0.05). In the 25–35 cm diameter class range, SW at heights of 0.3 m and 1.3 m shows significant differences when compared to the 0.8 m height (*p* < 0.05); HMC and SMC at 1.3 m exhibit significant differences compared to the other two heights (*p* < 0.05). In the 35–45 cm diameter class, HD, HMC, and SMC at 1.3 m all show significant differences from the other two heights (*p* < 0.05). For diameters greater than 45 cm, no significant differences are observed across the indicators (*p* > 0.05).

Regarding the variation patterns of heartwood and sapwood indicators at different sampling heights, the following trends are observed: At 0.3 m height, SD in the 15–25 cm and 25–35 cm diameter classes does not differ significantly (*p* > 0.05), but significant differences are observed when compared to the other two diameter classes (*p* < 0.05); HMC shows significant differences across all four diameter classes (*p* < 0.05); and SMC shows no significant differences in the 35–45 cm and greater than 45 cm diameter classes (*p* > 0.05), but significant differences are noted between the other two diameter classes (*p* < 0.05). At 0.8 m height, HD in the 15–25 cm and greater than 45 cm diameter classes does not show significant differences (*p* > 0.05), but significant differences are observed with the other two diameter classes (*p* < 0.05); SD and HMC show significant differences between the 15–25 cm and 25–35 cm diameter classes and the other two diameter classes (*p* < 0.05); and SMC shows significant differences between the 35–45 cm and greater than 45 cm diameter classes and the other two diameter classes (*p* < 0.05). At 1.3 m height, SW, HD, and H% show significant differences between the 15–25 cm and 35–45 cm diameter classes and the other two diameter classes (*p* < 0.05); SA and S% show significant differences within the 25–35 cm diameter class compared to the other three diameter classes (*p* < 0.05); SD shows significant differences between the 35–45 cm and greater than 45 cm diameter classes and the other two diameter classes (*p* < 0.05); HMC shows significant differences between the 15–25 cm and 25–35 cm diameter classes and the other two diameter classes (*p* < 0.05); and SMC shows significant differences across all diameter classes (*p* < 0.05).

### 2.2. Analysis of Soil Physical and Chemical Properties

#### 2.2.1. Characterization of Soil Water Salinity at Different Depths

**Figure 1:**
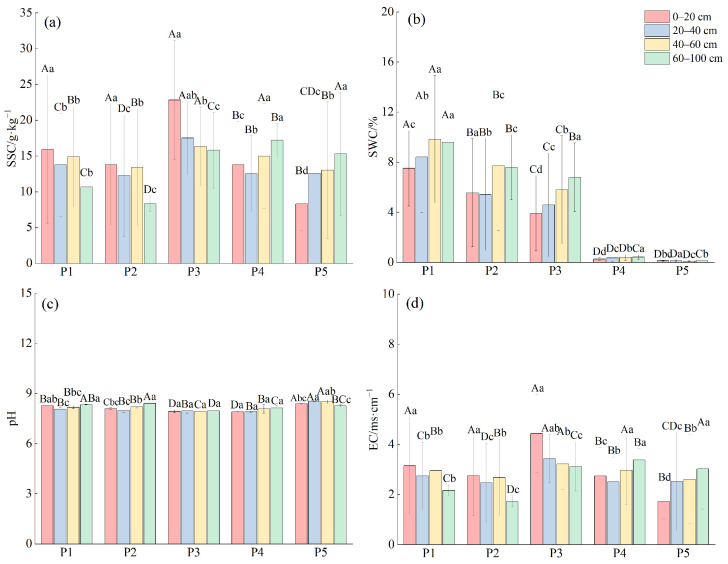
Characteristics in soil water and salt indicators at different depths in various plots. (**a**) SSC: soil salt content; (**b**) SWC: soil water content; (**c**) pH: acidity and alkalinity; (**d**) EC: electrical conductivity. Different capital letters in the figure indicate significant differences between sample plots, and different lowercase letters indicate significant differences between soil layers, *p* < 0.05.

Comparison of changes in soil water and salt indicators across sites (Figure 1). SWC in the study area ranged from 0.09% to 12.44%, with significant differences (*p* < 0.05) among soil layers at each sample site. The overall trend showed increasing SWC with depth. Soil pH ranged from 7.91 to 8.54, indicating alkaline conditions. No significant differences (*p* > 0.05) were found between the P3 and P4 horizons, but significant differences (*p* < 0.05) were observed between soil horizons at P1, P2, and P3. Soil EC ranged from 1.06 to 4.82 ms·cm^−1^, with corresponding salt content ranging from 4.76 g·kg^−1^ to 24.89 g·kg^−1^. Significant differences (*p* < 0.05) in EC and SSC were observed between horizons.

At P1, P2, and P3, EC and SSC tended to decrease with increasing soil depth, whereas at P4 and P5, they gradually increased with depth. Overall, SWC decreased as the distance from the river increased, while variations in pH, EC and SSC became more complex.

#### 2.2.2. Characterization of Soil Nutrients at Different Depths

**Figure 2:**
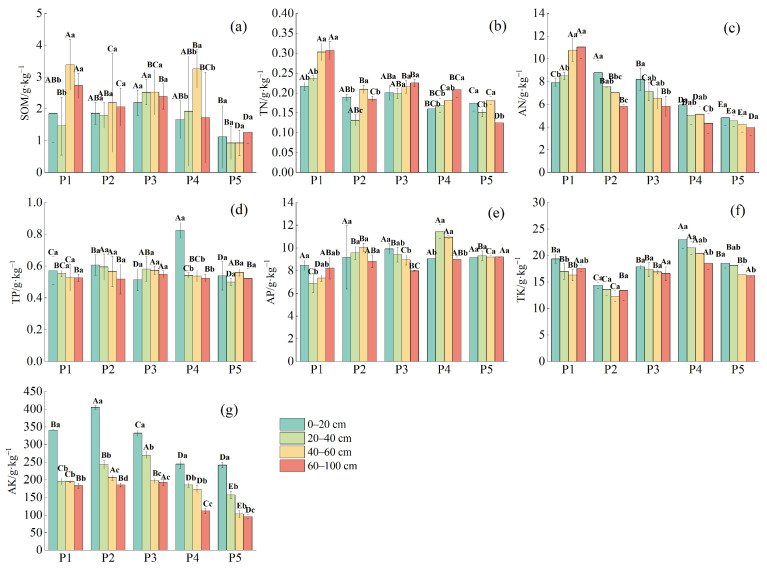
Characteristics in soil nutrient indicators at different depths in various plots. (**a**) SOM: soil organic matter; (**b**) TN: total nitrogen; (**c**) AN: alkaline hydrolysable nitrogen; (**d**) TP: total phosphorus; (**e**) AP: available phosphorus; (**f**) TK: total potassium; (**g**) AK: available potassium. Different capital letters in the figure indicate significant differences between sample plots, and different lowercase letters indicate significant differences between soil layers, *p* < 0.05.

The variation in soil nutrient indices across the different sample sites (Figure 2) indicates that SOM content ranged from 1.60 to 5.85 g·kg^−1^. Significant differences in SOM were observed between sample sites across different soil layers (*p* < 0.05), but overall, there was no clear trend of change with increasing soil depth. TN and AN content ranged from 0.12 to 0.31 g·kg^−1^ and 3.95 to 11.05 mg·kg^−1^, respectively. At site P3, the TN content across soil layers did not reach a significant level (*p* > 0.05), while significant differences were observed at the other sites and soil layers (*p* < 0.05). AN content did not reach a significant level (*p* > 0.05) at site P5, but significant differences were found at the other sites and soil layers (*p* < 0.05). TN content increased with soil depth at sites P1, P3, and P4, while AN increased with depth at site P1 but showed a decreasing trend at the other sites.

TP and AP contents ranged from 0.50 to 0.83 g·kg^−1^ and 6.89 to 11.46 mg·kg^−1^, respectively. At site P4, significant differences in TP were observed across soil layers (*p* < 0.05), with TP content generally decreasing with soil depth. AP decreased with depth at site P3 but showed no clear pattern in the other sites. TK and AK contents ranged from 12.31 to 23.02 g·kg^−1^ and 96.19 to 405.05 mg·kg^−1^, respectively. No significant differences in TK were found between soil layers at sites P2 and P3 (*p* > 0.05), but significant differences were observed at the other sites (*p* < 0.05). AK content showed significant differences across all sites and soil layers (*p* < 0.05). Overall, both TK and AK contents decreased with increasing soil depth.

#### 2.2.3. Principal Component Analysis of Soil Physical and Chemical Properties

**Figure 3:**
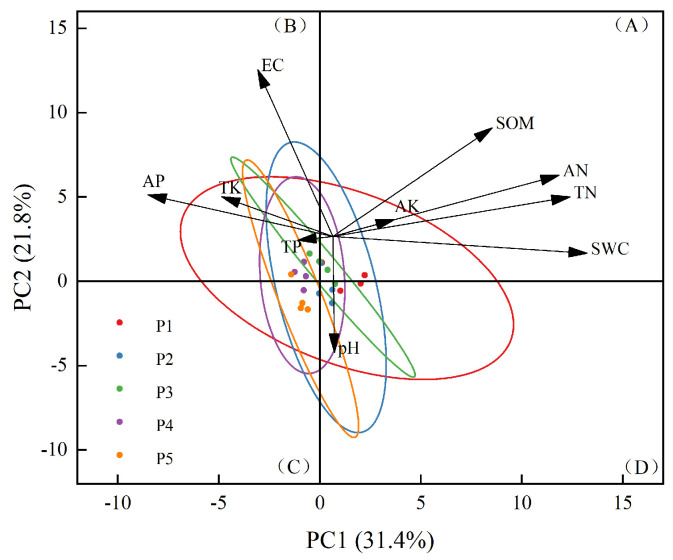
Principal component analysis of soil physical and chemical properties. The ellipses in the figure represent the 95% confidence zones of the soil physical and chemical property data for each site. The rest of the letters are the same as above.

The soil physicochemical properties varied significantly across the sample sites (Figure 3). The properties of P1 exhibited the largest horizontal axis span, with SWC and TN being the most influential factors, primarily distributed in area D. The properties of P2 showed the largest vertical axis span, predominantly distributed in areas B and D, with TP, EC, and SWC as the key influencing factors. In contrast, the properties of P3 had a smaller overall span and appeared to be more stable. The properties of P4 were mainly distributed in area B, with total potassium TP, AP, TK, and EC playing dominant roles. Meanwhile, the properties of P5 were primarily distributed in area C, where pH had the most significant influence.

These results highlight the distinct differences in soil physicochemical properties across the sample sites, which may be attributed to variations in the water table and soil type.

### 2.3. Relationship Between the Physical Characteristics of Heartwood and Sapwood in P. euphratica Trunks and the Physical and Chemical Properties of Soil

**Figure 4:**
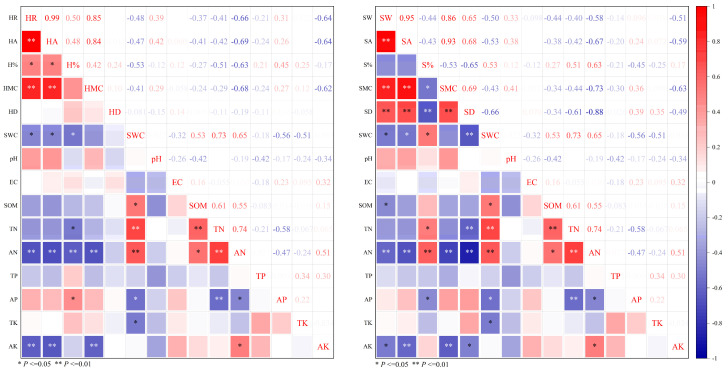
Correlation analysis of soil physicochemical properties with physical characteristics of heartwood and sapwood. ** and * indicate significant correlation at the 0.01 and 0.05 levels, respectively. The rest of the letters are the same as above.

Correlation analysis of physical characteristics of heartwood and sapwood with soil physico-chemical properties (Figure 4), soil TN exhibited a significant negative correlation with H % at the 0.05 level. AN was highly significantly negatively correlated with HR, HA, H% and HMC (*p* < 0.01), with correlation coefficients of 0.66, 0.69, 0.63, and 0.68, respectively. AP showed a positive correlation with H%, with a correlation coefficient of 0.51. AK was highly significantly negatively correlated with HR, HA and HMC at the 0.01 level, with correlation coefficients of 0.64, 0.64, and 0.62, respectively.

SOM was significantly negatively correlated with SW. TN exhibited a significant positive correlation with S% and a highly significant negative correlation with SD (*p* < 0.01), while it was highly significantly positively correlated with SMC (*p* < 0.01). AN was highly significantly negatively correlated with SW, SA, SMC and SD (*p* < 0.01), while it was highly significantly positively correlated with S% at the 0.01 level. Soil AP was significantly negatively correlated with S% and SMC. TK was significantly negatively correlated with SMC. AK was significantly negatively correlated with SW and SD, and highly significantly negatively correlated with SA and SMC (*p* < 0.01). SWC was highly significantly negatively correlated with SW, SA and SD (*p* < 0.01), while it showed a significant positive correlation with S% (*p* < 0.05).

**Figure 5:**
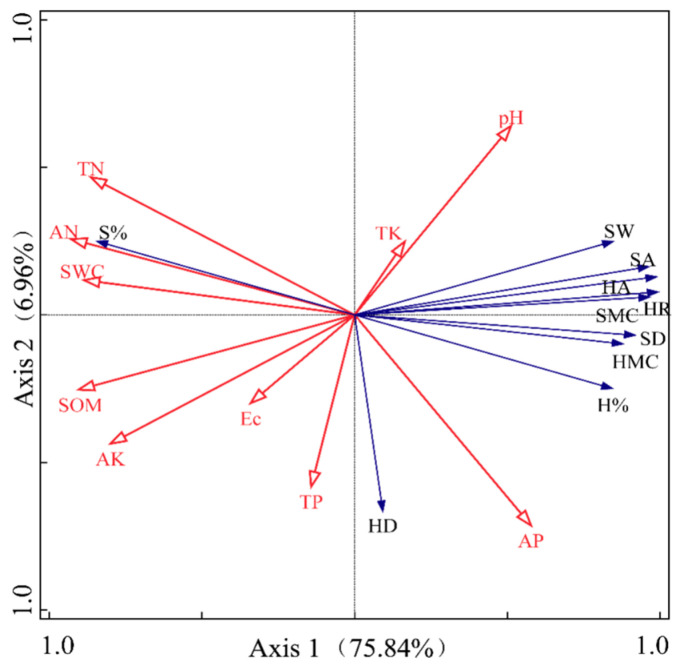
RDA analysis for physical characteristics of heartwood/sapwood and its influencing factors.

Based on the RDA analysis of the physical characteristics of heartwood and sapwood in *P. euphratica* trunks in relation to influencing factors at each sample site (Figure 5), the first and second axes explained 82.80% of the total variance. This indicates that the first two axes effectively captured the relationship between the physical traits of heartwood and sapwood and the influencing factors, with the first axis being the most influential. The results showed that AN, SWC and TN had the greatest impact on S%, with positive correlations, while these factors were negatively correlated with SW, SA, SMC, SD, HR, HA, H%, and HMC. Soil pH and AP were positively correlated with SW, SA, SMC, SD, HR, HA, H%, and HMC. In contrast, AK and SOM were negatively correlated with these same traits. TP had the greatest influence on HD, showing a positive correlation. Furthermore, among the environmental factors, SWC, pH, SOM, TN, AN, AK, and AP had the most significant impact on the physical characteristics of *P. euphratica* heartwood and sapwood.

## 3. Discussion

### 3.1. Changing Patterns in the Physical Properties of Heartwood and Sapwood in P. euphratica Trunks

In arid regions, *P. euphratica* adapts to environmental heterogeneity by regulating the dynamic relationship between heartwood and sapwood. As heartwood gradually forms from the transformation of sapwood, its distribution pattern reflects the tree’s physiological responses and growth strategies during development [17,18]. This study found that the radius, area, and proportion of heartwood gradually decreased from the base to the top of the trunk, consistent with the process of sapwood transitioning into heartwood. This pattern aligns with the findings of Luo et al. (2021) [19] in Eucalyptus grandis. Furthermore, environmental factors, such as the distance from the river, play a crucial role in shaping the physical properties of both heartwood and sapwood. Our analysis shows that as the distance from the river increases, significant changes occur in the structure and composition of both heartwood and sapwood. Specifically, trees closer to the river exhibit higher proportions and densities of heartwood, which can be attributed to the more abundant water supply and stable environmental conditions in these areas. In contrast, trees located farther from the river display lower proportions of heartwood, indicating a shift in resource allocation in response to more variable water availability and harsher environmental conditions. These findings highlight the tree’s adaptive strategy in optimizing structural integrity and water transport capacity across different environmental gradients [20,21,22]. We propose that the distribution of heartwood and sapwood is not only a structural adaptation for water transport but also reflects an evolutionary strategy for the tree to cope with arid conditions [23,24,25]. Specifically, this adaptation could be an example of phenotypic plasticity, where *P. euphratica* adjusts its growth and physiological processes in response to varying environmental factors, rather than through evolutionary changes alone [26].

In the early stages of growth, sapwood plays a crucial role in water conduction, which is vital for the tree’s survival in arid environments. However, as the tree matures, the formation of heartwood provides structural support [27]. The increase in sapwood as the tree grows is likely to support these physiological needs, while the increase in heartwood is associated with the aging and eventual death of sapwood cells. This transition from sapwood to heartwood can be seen as an example of phenotypic plasticity, where the tree modifies the distribution and properties of heartwood and sapwood to optimize its structural integrity and water transport capacity as it ages [28,29].

A comparison of different DBH classes of *P. euphratica* showed that heartwood water content was significantly higher than that of sapwood, suggesting that heartwood may store water under certain environmental conditions [30]. However, further research is needed to confirm whether heartwood plays a significant role in long-term water storage, especially considering the relationship between water content and overall tissue volume or density. We hypothesize that this water retention capacity in heartwood could be an adaptive response to fluctuating water availability in arid regions, although its role in long-term storage remains unclear. The role of heartwood in water storage is likely influenced by its dense structure, which may temporarily retain water absorbed during rainfall events, but not actively transport it.

In the early stages of tree growth, heartwood density was higher than sapwood density, likely due to the loss of physiologically active cells in the heartwood, leading to a relative increase in water retention in the heartwood [31]. Although heartwood does not actively conduct water, it may store some due to its compact structure. As the tree matures, heartwood density increases, and its water retention capacity decreases, reflecting the cessation of metabolic activity in heartwood cells. This transition marks the shift from water conduction in sapwood to the structural support role of heartwood, underscoring the dynamic adaptation of *P. euphratica* to dry environments and the varying conditions along the river gradient [32,33].

### 3.2. Influence of Soil Physicochemical Properties on the Physical Characteristics of Heartwood and Sapwood in P. euphratica Trunks

Soil physicochemical properties are fundamental environmental factors influencing plant growth and play a crucial role in the development of tree trunks, including heartwood and sapwood formation [7]. The study area’s alkaline soil, characterized by heavy salinization, may impact the growth and functional development of the *P. euphratica* root system, thus limiting tree growth. In this study, soil water content generally increased with soil depth, consistent with the vertical increment law of soil moisture. However, other soil properties, such as pH, organic matter, and quick-acting phosphorus, did not show a clear vertical pattern, while conductivity and salinity tended to decrease with soil depth [34]. Nutrients such as total phosphorus, total potassium, and quick-acting potassium also exhibited a vertical decreasing trend, likely due to the movement of water in the soil affecting nutrient distribution. The topsoil is more susceptible to rainfall and irrigation, which can leach dissolved nutrients, resulting in lower nutrient content at the surface. Soil moisture significantly impacts *P. euphratica* population structure and growth [35].

The correlation analysis results show that the physical characteristics of *P. euphratica* sapwood in the lower reaches of the Tarim River are greatly influenced by environmental factors. Specifically, the physicochemical properties of the soil layers exhibit a strong correlation with various indices of sapwood physical characteristics, which may be related to the distribution of the tree’s root system [36]. Factors such as the water-salt balance and nutrient conditions in deeper soil layers have a more pronounced effect on *P. euphratica* growth, establishing a closer relationship with certain physical traits of the heartwood and sapwood. Additionally, soil organic matter not only impacts the growth of the heartwood and sapwood but also influences the overall growth condition of the trees. Redundancy analysis (RDA) results indicate that the first axis provides the highest explanatory power when exploring the relationships between the physical characteristics of heartwood and sapwood and influencing factors across various sites. This suggests that the primary environmental factors driving changes in physical traits are closely associated with variations along the first axis. Under extreme drought conditions, *P. euphratica* adapts by increasing its trunk’s water-conducting capacity and the degree of xylem embolism [37]. The xylem, composed of heartwood and sapwood, plays a vital role in water transport. Changes in the physical characteristics of both heartwood and sapwood are part of the tree’s adaptation to drought stress.

The effects of soil moisture, salinity, and nutrients on the physical properties of heartwood and sapwood are crucial for understanding the ecological adaptations of poplar trees. Soil moisture, for instance, can greatly influence the water content in *P. euphratica* sapwood, thereby enhancing its ability to transfer water efficiently. Conversely, changes in soil salinity and pH levels can modulate the growth rate, drought tolerance, and overall physiological responses of the tree. Kint’s observed a minimal impact of soil moisture on the heartwood of Quercus europaea, which contrasts with our findings for *P. euphratica* [38]. This discrepancy may stem from the different ecological contexts of the two studies. In our research, soil moisture is a critical factor influencing plant growth, and water stress significantly affects the physiological activity of sapwood cells. Thin-walled sapwood cells are particularly sensitive to water availability, and this stress can influence heartwood formation. As a result, *P. euphratica* heartwood and sapwood are more directly affected by water content compared to Quercus europaea. Organic matter, a key indicator of soil fertility, also plays a significant role in poplar growth, particularly in affecting the heartwood’s physical properties. This finding is consistent with Bektas et al. (2003) [39], who highlighted the importance of organic matter in tree growth and wood characteristics. Nutrients such as potassium and phosphorus also influence the physical properties of *P. euphratica* wood. Potassium is essential for several physiological processes, including enzyme activation, photosynthesis, carbohydrate metabolism, and protein synthesis [9]. Its availability directly impacts the function of sapwood, which subsequently influences the formation of heartwood. Likewise, phosphorus, as identified by Wright et al. (2011) [40] as a key nutrient for forest growth and wood yield, plays an essential role in energy storage, cell division, and root development. For *P. euphratica*, phosphorus is vital for forming strong root systems, which are necessary for water and nutrient uptake in drought-stressed environments. The ability of *P. euphratica* to transport water and nutrients through sapwood helps maintain the physical integrity of both the heartwood and sapwood. In summary, soil moisture, organic matter, and key nutrients like potassium and phosphorus play interconnected roles in shaping the physical properties of *P. euphratica* heartwood and sapwood, helping the tree adapt to environmental stresses such as water scarcity.

In view of the above analyses, this paper puts forward the directions worth exploring and researching in the future: (1) Focus on the accuracy and comprehensiveness of the sampling method. In this study, *P. euphratica* as a protected tree species cannot be cut down for destructive sampling, in the future with the help of instrumentation for more accurate and comprehensive sampling; (2) focus on the comprehensiveness of soil sampling. In the future, the soil sampling can be analysed according to the season, so as to comprehensively study the influence of soil physicochemical factors on the growth of poplar; (3) this paper mainly focuses on the analysis of the physical characteristics of *P. euphratica* heartwood and sapwood, and it is recommended to add the *P. euphratica*’s chemical characteristics, ecological stoichiometry and other related contents to do a comprehensive investigation on the growth of *P. euphratica* in the future study.

## 4. Materials and Methods

### 4.1. Overview of the Study Area

**Figure 6:**
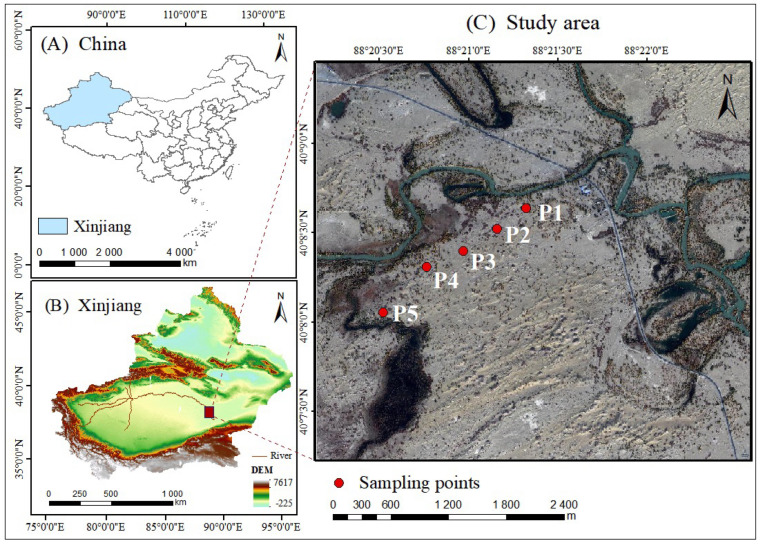
Overview map of the study area.

The study area is located in the Arghan region of the lower Tarim River, Xinjiang, China (Figure 6). The maximum temperatures can reach 43.6 °C, with a mean annual temperature of 10.8 °C. The region experiences low mean annual precipitation, with a range of 17.4–42.0 mm, while evapotranspiration is extremely high, with a multi-year average potential evapotranspiration of 2500–3000 mm [14]. The mean annual precipitation is significantly lower than the evapotranspiration rate. The vegetation in the region mainly consists of trees, shrubs, and herbaceous plants, with *P. euphratica* serving as the dominant species in the lower reaches of the Tarim River. The accumulation of salts from water bodies in surface soil results in the formation of extensive saline soils. Most river terraces are composed of *P. euphratica* forest meadow soils, whereas, areas situated at a greater distance from the river tend to evolve into wind-sand soils or salt-alkali soils [41]. As the distance from the river increases, particularly in the downstream, the proportion of wind-sand soils rises increases, resulting in significant desertification and salinization across the entire watershed [42].

### 4.2. Sample Plot Setup and Data Acquisition

Five sample plots were established at varying distances from the river, with standard 50 m × 50 m plots located at P1 (50 m), P2 (300 m), P3 (500 m), P4 (750 m), and P5 (1050 m) from the river (Table 2). DBH was used as a proxy for age class [43], with the poplars in the sample plots were classified into four DBH groups: 15–25 cm, 25–35 cm, 35–45 cm, and greater than 45 cm. Wherever feasible, three healthy *P. euphratica*, representing disparate DBH classes, were selected from each sample plot. The trees selected exhibited robust growth, straight trunks, and no signs of pest or disease infestation. Given the existence of variations in the predominant stand types across the sample plots, the number of samples collected within each plot differed. A total of 42 sample trees were selected, and the altitude, latitude, and longitude of each tree were recorded using GPS. DBH was measured by using a diameter-at-breast ruler, with an error margin of less than 0.1 cm. To minimize potential parallax errors and inaccuracies due to irregularities in tree bole shape, DBH was measured at multiple points around the tree’s circumference and averaged. Tree height (TH) was measured using a Bloom-Rice altimeter.

Tree cores were extracted from the selected *P. euphratica* samples at different heights (0.3 m, 0.8 m, 1.3 m) using tree increment borers with a diameter of 5.15 mm to differentiate between heartwood and sapwood. The bark thickness (BT) and sapwood width (SW) were then measured using vernier calipers, as reported in the results:

(1) $$\mathrm{HR}=\frac{\mathrm{DBH}}{2}-\mathrm{BT}-\mathrm{SW}$$

(2) $$\mathrm{HA}={\pi \mathrm{HR}}^{2}$$

(3) $$\mathrm{SA}=\pi {\left(\frac{\mathrm{DBH}}{2}-\mathrm{BT}\right)}^{2}-\mathrm{HA}$$

The sample cores were returned to the laboratory, where the fresh weights were measured using an electronic balance with an accuracy of 0.0001 g. The fresh weights of heartwood and sapwood were recorded as m1 and m2, respectively, while their dry masses were recorded as m3 and m4, respectively. The heartwood moisture content (HMC) and sapwood moisture content (SMC) were calculated as follows:

(4) $$\mathrm{HMC}=\frac{\left(m3-m1\right)}{m1}\times 100\%$$

(5) $$\mathrm{SMC}=\frac{\left(m4-m2\right)}{m2}\times 100\%$$

The lengths of the heartwood and sapwood were recorded as L1 and L2, respectively, and their volumes were calculated as V1 and V2. The basic density of the heartwood (HD) and sapwood (SD) was then determined using the following formula:

(6) $$\mathrm{HD}=\frac{m3}{V1}$$

(7) $$\mathrm{SD}=\frac{m4}{V2}$$

(8) $$H\%=\frac{\mathrm{HA}}{\mathrm{Peeled}\;\mathrm{cross}-\mathrm{sectional}\;\mathrm{area}}$$

(9) $$S\%=\frac{\mathrm{SA}}{\mathrm{Peeled}\;\mathrm{cross}-\mathrm{sectional}\;\mathrm{area}}$$

### 4.3. Soil Sample Collection and Processing

Three 1 m × 1 m sample plots were established along the diagonal, with each plot excavated to a depth of 1 m. Soil samples were collected from four discrete soil horizons: 0–20 cm, 20–40 cm, 40–60 cm, and 60–100 cm. The samples were taken in aluminum boxes and brought to the laboratory for analysing moisture content. Subsequently, the soil was backfilled into the pits in a sequential manner. For each depth, the soil samples were subjected to air-drying and sieving through a 2 mm mesh. Subsequently, a range of soil physicochemical properties were analyzed using the following methods, as outlined by [44]: soil moisture content: drying method; soil organic matter: potassium dichromate heating method; soil total nitrogen: semi-micro Kjeldahl method; soil alkaline dissolved nitrogen: alkaline diffusion method; soil total phosphorus and available phosphorus: sodium bicarbonate leaching, followed by molybdenum-antimony colorimetric method; soil total potassium: alkali fusion and flame photometric method; soil available potassium: ammonium acetate leaching and flame photometric method.

To guarantee the independence of the data, the soil electrical conductivity (EC), soil salt content (SSC), and pH were measured separately for each depth sample, thus preventing cross-contamination during the analysis. Soil Electrical Conductivity (EC): a known volume of soil from each depth was combined with deionized water in a 1:5 ratio. The mixture was then left to stand at room temperature for 30 min, with gentle shaking every 10 min to ensure complete dissolution of salts. Subsequently, the conductivity of the solution was determined using a conductivity meter. The soil salt content (SSC): A dry soil sample from each depth was mixed with deionized water at a ratio of 1:10. The mixture was then shaken thoroughly and left to stand for 24 h. Following this period, the salt content was measured. Soil pH: for each depth, soil samples were mixed with deionized water at a 1:2.5 ratio. The mixture was then left to rest for one hour at room temperature to ensure sufficient dissociation of hydrogen ions from the soil. The pH was then measured using a glass electrode pH meter.

### 4.4. Data Processing and Analysis

The data on heartwood, sapwood, and soil physico-chemical indicators were compiled using the Microsoft Excel 2019. Normality testing and data transformation were conducted using SPSS 26.0, followed by one-way ANOVA and multiple comparisons (LSD-*t* test) to analyze the differences in the physical characteristics of heartwood and sapwood at varying heights, with a significance level of 0.05. Principal component analysis was employed to examine soil physico-chemical properties. Pearson’s correlation analysis was conducted using a two-tailed significance test, with a level of significance at 0.05. The generation of graphs was conducted using the Origin 2021 software.

## 5. Conclusions

*P. euphratica* showed remarkable adaptability in extreme arid environments, optimizing water storage and conductance under arid conditions by varying the water content and density of heartwood and sapwood. It was found that the water content of the heartwood ranged from 25.65% to 44.46%, which was much higher than that of the sapwood ranging from 13.65% to 30.58%, showing its remarkable water utilisation mechanism. The soil moisture content of the near-river sample (e.g., P1, 50 m from the river) was as high as 12.44%, while that of the sample far from the river (e.g., P5, 1050 m from the river) was only 0.09%. This moisture difference may have directly affected the heart and sapwood characteristics of *P. euphratica* plants. The heartwood density was higher in young *P. euphratica* (0.35–0.42 g/cm^3^) and gradually became lower than the sapwood (0.36–0.44 g/cm^3^) as the tree aged, reflecting adaptive changes in *P. euphratica*. Taken together, SWC, pH, SOM, TN, AN, AK, and AP had a greater influence on the physical characteristics of *P. euphratica* heart sapwood, which were similar to the limiting factors of *P. euphratica* growth. The response of *P. euphratica* to environmental factors was not only reflected in the growth indicators, but also in the changes of physical characteristics of heartwood and sapwood, reflecting the self-regulation ability of *P. euphratica* to the changes in the growth environment. Considering the reliance of *P. euphratica* growth on soil water content and nutrients, it was recommended to enhance soil quality by increasing the levels of organic matter, nitrogen, and phosphorus, particularly in arid regions, to improve the drought resilience and ecological restoration capacity of *P. euphratica* forests. This study revealed the ecological adaptability of *P. euphratica* in extreme environments and provided guidance for ecological management practices.

## Figures and Tables

**Table 1:** Differences between heartwood and sapwood indicators.

Index		DBH	15–25 cm	25–35 cm	35–45 cm	>45 cm	Average
	Height						
HR	0.3 m		8.64 ± 1.62 Da	11.09 ± 1.58 Ca	15.63 ± 1.82 Ba	18.91 ± 0.87 Aa	12.80 ± 4.01
	0.8 m		8.33 ± 1.94 Da	10.95 ± 0.75 Ca	14.96 ± 1.96 Ba	18.90 ± 1.17 Aa	12.48 ± 3.99
	1.3 m		7.93 ± 1.36 Da	11.15 ± 0.90 Ca	15.08 ± 1.55 Ba	18.34 ± 1.00 Aa	12.37 ± 3.87
SW	0.3 m		1.95 ± 0.44 Ca	2.53 ± 0.56 Ba	2.73 ± 0.40 ABa	3.00 ± 0.16 Aa	2.49 ± 0.57
	0.8 m		1.91 ± 0.33 Ca	2.27 ± 0.42 Cab	2.69 ± 0.63 Ba	3.17 ± 0.08 Aab	2.42 ± 0.61
	1.3 m		1.82 ± 0.39 Ca	2.11 ± 0.25 Cb	2.48 ± 0.54 Ba	3.30 ± 0.21 Ab	2.30 ± 0.61
HA	0.3 m		242.15 ± 84.49 Da	393.77 ± 112.92 Ca	776.52 ± 180.59 Ba	1125.06 ± 104.18 Aa	526.57 ± 316.31
	0.8 m		228.93 ± 100.30 Da	378.15 ± 52.08 Ca	714.31 ± 178.49 Ba	1125.96 ± 141.67 Aa	538.39 ± 330.98
	1.3 m		203.14 ± 66.10 Da	392.58 ± 62.95 Ca	721.64 ± 144.68 Ba	1058.74 ± 116.72 Aa	526.57 ± 316.31
SA	0.3 m		120.30 ± 40.87 Da	198.78 ± 60.30 Ca	293.35 ± 65.40 Ba	383.97 ± 20.87 Aa	229.83 ± 105.24
	0.8 m		111.83 ± 29.05 Da	172.49 ± 32.57 Cab	278.49 ± 85.76 Ba	408.02 ± 26.27 Aa	219.09 ± 113.26
	1.3 m		100.67 ± 29.18 Da	162.04 ± 24.33 Cb	255.97 ± 62.73 Ba	409.98 ± 42.36 Aa	209.18 ± 112.98
HD	0.3 m		0.35 ± 0.04 Aa	0.39 ± 0.06 Aa	0.38 ± 0.04 Aa	0.37 ± 0.01 Aa	0.37 ± 0.02
	0.8 m		0.37 ± 0.04 Ab	0.41 ± 0.05 Aa	0.39 ± 0.03 Aab	0.37 ± 0.01 Ab	0.38 ± 0.02
	1.3 m		0.38 ± 0.02 Cc	0.42 ± 0.03 Aa	0.39 ± 0.02 BCbc	0.4 ± 0.03 ABab	0.4 ± 0.02
SD	0.3 m		0.37 ± 0.05 Cb	0.38 ± 0.04 BCb	0.44 ± 0.04 Aa	0.43 ± 0.01 ABa	0.4 ± 0.04
	0.8 m		0.36 ± 0.04 Bc	0.39 ± 0.05 ABbc	0.42 ± 0.03 Aab	0.44 ± 0.01 Aa	0.4 ± 0.04
	1.3 m		0.36 ± 0.02 Cc	0.4 ± 0.03 Bb	0.43 ± 0.01 Aa	0.44 ± 0.01 Aa	0.41 ± 0.03
HMC	0.3 m		27.53 ± 2.93 Dd	31.97 ± 3.13 Cc	39.64 ± 6.3 Bb	44.46 ± 1.23 Aa	35.9 ± 7.59
	0.8 m		26.93 ± 3.76 Cc	30.13 ± 4.1 Cc	36.87 ± 4.89 Bb	42.31 ± 3.21 Aa	34.06 ± 6.88
	1.3 m		28.63 ± 1.44 Bb	28.14 ± 2.12 Bb	34.87 ± 5.73 Aa	37.51 ± 3.49 Aa	32.29 ± 4.64
SMC	0.3 m		13.65 ± 1.72 Cc	18.64 ± 6.17 Bb	25.58 ± 3.03 Aa	28.31 ± 0.99 Aa	21.54 ± 6.65
	0.8 m		13.8 ± 0.99 Cc	17.45 ± 3.47 Bb	25.61 ± 3.25 Aa	27.97 ± 0.26 Aa	21.23 ± 6.65
	1.3 m		14.11 ± 0.83 Cd	17.81 ± 3.58 Cc	24.84 ± 0.77 Bb	30.58 ± 2.15 Aa	21.84 ± 7.34
H%	0.3 m		0.66 ± 0.05 Ba	0.66 ± 0.06 Ba	0.72 ± 0.03 Aa	0.74 ± 0.02 Aa	0.69 ± 0.06
	0.8 m		0.65 ± 0.07 Ba	0.69 ± 0.05 ABab	0.72 ± 0.05 Aa	0.73 ± 0.01 Aab	0.69 ± 0.06
	1.3 m		0.66 ± 0.06 Ba	0.71 ± 0.03 ABb	0.74 ± 0.06 Aa	0.72 ± 0.02 Ab	0.70 ± 0.06
S%	0.3 m		0.34 ± 0.05 Aa	0.34 ± 0.06 Aa	0.28 ± 0.03 Ba	0.26 ± 0.02 Bb	0.31 ± 0.06
	0.8 m		0.35 ± 0.07 Aa	0.31 ± 0.05 ABab	0.28 ± 0.05 Ba	0.27 ± 0.01 Bab	0.31 ± 0.06
	1.3 m		0.34 ± 0.06 Aa	0.29 ± 0.03 ABb	0.26 ± 0.06 Ba	0.28 ± 0.02 Ba	0.30 ± 0.06

Note: Data are mean ± standard deviation. HR: Heartwood Radius; SW: Sapwood Width; HA: Heartwood Area; SA: Sapwood Area; HD: Heartwood Density; SD: Sapwood Density; HMC: Heartwood Moisture Content; SMC: Sapwood Moisture Content; H%: Heartwood Rate; S%: Sapwood Rate. Different uppercase letters are significant between diameters, and lowercase letters are significant between heights.

**Table 2:** Summary of sampling plots in the study area.

Plot	Distance from River/m	Longitude and Latitude	Elevation Level/m	Types of Stands	Average DBH/cm	Average TH/m
P1	50	40°08′10″, 88°22′48″	820	middle-aged forest	22.11 ± 7.82	6.42 ± 2.29
P2	300	40°08′11″, 88°22′34″	818	middle-aged forest	26.18 ± 0.09	6.67 ± 2.16
P3	500	40°08′08″, 88°22′04″	818	middle-aged forest	21.25 ± 8.91	7.73 ± 4.39
P4	750	40°08′03″, 88°21′56″	820	mature forest	31.52 ± 7.68	9.8 ± 5.2
P5	1050	40°07′56″, 88°21′47″	821	overmature forest	47.58 ± 4.12	5.42 ± 1.47

Note: Data are mean ± standard deviation.

## Data Availability

The data that support the findings of this study are available from the corresponding author on reasonable request.
